# Diversity of the Tellurite Resistance Gene Operon in *Escherichia coli*

**DOI:** 10.3389/fmicb.2021.681175

**Published:** 2021-05-28

**Authors:** Thi Thu Huong Nguyen, Taisei Kikuchi, Tadaaki Tokunaga, Sunao Iyoda, Atsushi Iguchi

**Affiliations:** ^1^Department of Environment and Resource Sciences, University of Miyazaki, Miyazaki, Japan; ^2^Thai Nguyen University of Agriculture and Forestry, Thai Nguyen, Vietnam; ^3^Department of Infectious Disease, Faculty of Medicine, University of Miyazaki, Miyazaki, Japan; ^4^Department of Bacteriology I, National Institute of Infectious Diseases, Tokyo, Japan

**Keywords:** *ter* operon, tellurite resistance, Shiga toxin-producing *Escherichia coli*, integrating genetic element, horizontal genetic transfer

## Abstract

Tellurite is highly toxic to most bacteria owing to its strong oxidative ability. However, some bacteria demonstrate tellurite resistance. In particular, some *Escherichia coli* strains, including Shiga toxin-producing *E. coli* O157:H7, are known to be resistant to tellurite. This resistance is involved in *ter* operon, which is usually located on a prophage-like element of the chromosome. The characteristics of the *ter* operon have been investigated mainly by genome analysis of pathogenic *E. coli*; however, the distribution and structural characteristics of the *ter* operon in other *E. coli* are almost unknown. To clarify these points, we examined 106 *E. coli* strains carrying the *ter* operon from various animals. The draft genomes of 34 representative strains revealed that *ter* operons were clearly classified into four subtypes, *ter*-type 1–4, at the nucleotide sequence level. Complete genomic sequences revealed that operons belonging to three *ter*-types (1, 3, and 4) were located on the prophage-like elements on the chromosome, whereas the *ter*-type 2 operon was located on the IncHI2 plasmid. The positions of the tRNA^Ser^, tRNA^Met^, and tRNA^Phe^ indicated the insertion sites of elements carrying *the ter* operons. Using the PCR method developed in this study, 106 strains were classified as type 1 (*n* = 66), 2 (*n* = 13), 3 (*n* = 8), and 4 (*n* = 17), and two strains carried both types 1 and 2. Furthermore, significant differences in the minimum inhibitory concentration (MIC) of tellurite were observed between strains carrying *ter*-type 4 and the others (*p* < 0.05). The *ter*-type was also closely related to the isolation source, with types 2 and 4 associated with chickens and deer, respectively. This study provided new insights related not only to genetic characteristics of the *ter* operons, but also to phenotypic and ecological characteristics that may be related to the diversity of the operon.

## Introduction

Tellurium (Te) is a rare element on Earth (Ibers, 2009) and is found in metal ores such as gold and copper (Terziev, 1966; Adams, 2016). Tellurite (TeO_3_^2–^), the oxyanion of tellurium, is highly toxic to most bacteria owing to its strong oxidative ability. Tellurite toxicity is related to the generation of reactive oxygen species (ROS) (Turner et al., 2001; Pérez et al., 2007; Tremaroli et al., 2007; Calderón et al., 2009; Díaz-Vásquez et al., 2014; Sandoval et al., 2015), which then induce damage to key cell components (Cabiscol et al., 2000; Imlay, 2003; Ling and Söll, 2010; Molina-Quiroz et al., 2013), and consequently affect bacterial growth. However, some bacteria demonstrate tellurite resistance. In particular, most Shiga toxin-producing *Escherichia coli* (STEC) belonging to O157:H7 and other serotypes such as O26:H11, O103:H2, O111:H8, O121:H19, O145:H28, and O45:H2, which are responsible for numerous foodborne disease outbreaks, are known to possess tellurite resistance. Therefore, when STEC is efficiently isolated from patient and food samples, selective agar plates containing potassium tellurite (K_2_TeO_3_), such as cefixime-tellurite sorbitol MacConkey (CT-SMAC) agar and CHROMagar STEC are routinely used in laboratories.

Several genes involved in arsenic efflux (*arsABC*) (Turner et al., 1992), cysteine metabolism (*cysK*, *cysM*, *csdB*, *IscS*) (Vásquez et al., 2001; Lithgow et al., 2004; Rojas and Vásquez, 2005), nitrate reduction (*narGHIJ*) (Avazéri et al., 1997; Sabaty et al., 2001), superoxide dismutation (*sodA*, *sodB*, *soxS*) (Tantaleán et al., 2003; Pérez et al., 2007), hydrogen peroxide detoxification (*katG*) (Calderón et al., 2006), tellurite resistance proteins (*tehAB, kilA, telAlB*) (Walter et al., 1991; Turner et al., 1995), and glucose-phosphate dehydrogenase (*zwf*) (Sandoval et al., 2011; Sandoval et al., 2015) have been found to be related with tellurite resistance. Additionally, the tellurite resistance gene (*ter*) operon (Whelan et al., 1995) is involved in tellurite resistance at relatively high concentrations; the *ter* operon was first identified from a conjugative plasmid of an *Alcaligenes* strain (Jobling and Ritchie, 1987, 1988). Although the detailed functions of the *ter* operon underlying tellurite resistance are not fully understood, experiments such as cloning have revealed that six genes (*terZABCDE*) are directly involved in tellurite resistance (Whelan et al., 1995; Kormutakova et al., 2000; Valková et al., 2007). Genome sequences deposited in databases have revealed that the *ter* operon is widely distributed in gram-negative bacteria including *Escherichia*, *Salmonella*, *Yersinia, Pseudomonas*, and *Proteus* species (Supplementary Tables 1, 2).

STECs belonging to the seven major serotypes described above are known to carry the *ter* operon. For example, the STEC O157:H7 Sakai strain carried the *ter* operon on a prophage-like 86-kb element, named SpLE1 on the chromosome (Hayashi et al., 2001; Ohnishi et al., 2002). SpLE1 also contains genes encoding integrase, urease (*ure*), and iron-regulated gene A (IrgA)-homolog adhesin (*iha*) (Hayashi et al., 2001). Comparative genome analyses indicated that the *ter* operons and their carrier elements are highly conserved among STEC strains belonging to the major serotypes (Ogura et al., 2009; Lorenz et al., 2017). Some STEC isolates, such as STEC O104:H4 and O178:H19, and non-STEC *E. coli* isolates also carry *ter* operons and/or show significant tellurite resistance (Orth et al., 2007; Gouali et al., 2013; Miko et al., 2014; Ferdous et al., 2015; Verhaegen et al., 2015; Kalule et al., 2018; Nguyen et al., 2021). A uropathogenic *E. coli* isolate was found to carry the *ter* operon on a large conjugative plasmid (Soltys et al., 2018). These reports suggest that the *ter* operon is widely distributed in *E. coli* including intestinal and extraintestinal pathogenic *E. coli*, as well as non-pathogenic *E. coli*. However, except for some STECs, the genetic characteristics of *ter* operons and their carrier elements have not been investigated in detail. In this study, in addition to STECs belonging to seven major serotypes, detailed analysis of *ter* operons and their carrier elements were performed in *E. coli* isolates belonging to more than 70 serotypes isolated from humans, cattle, pigs, chickens, and deer.

## Materials and Methods

### *E. coli* Strains

In total, 106 *terA*-positive *E. coli* strains isolated in Japan were used in this study (Supplementary Table 3). Human-derived STECs were isolated from patients and asymptomatic carriers through routine laboratory inspection. Twenty-four cattle-derived STEC strains, isolated in our previous study, were also used (Nguyen et al., 2021). Other *E. coli* strains from cattle, pigs, and deer were isolated from the rectal feces of slaughtered healthy animals. Feces were pre-cultured in modified *E. coli* broth (mEC) (Nissui Pharmaceutical Co., Ltd., Tokyo, Japan) at 37°C for 18–20 h and then inoculated on deoxycholate hydrogen sulfide lactose (DHL) (Nissui Pharmaceutical Co., Ltd., Tokyo, Japan) or CHROMagar STEC (CHROMagar^TM^ Paris, France) agar plates. *E. coli* strains from chickens were isolated from colibacillosis infected sites (such as under the skin and organs) of chickens on farms and slaughterhouses. The swabs were directly inoculated onto DHL agar plates. After incubation at 37°C for 18–20 h, *E. coli*-like single colonies were picked from each plate. DNA for PCR was purified using the Wizard^®^ Genomic DNA Purification Kit (Promega, Madison, WI, United States). *E. coli* species were confirmed by PCR using *E. coli*-specific *gyrB* sequences (Iguchi et al., 2015). The presence of the *ter* operon was screened by PCR using a universal primer pair (Supplementary Table 4), designed based on the *terA* sequences collected from the DNA database (GenBank/ENA/DDBJ) (Supplementary Table 1). The thermal conditions for PCR included 25 cycles of 94°C for 20 s, 58°C for 20 s, and 72°C for 30 s. PCR was conducted using Kapa Taq DNA polymerase (Kapa Biosystems, Woburn, MA, United States) or Dream Taq DNA polymerase (Thermo Fisher Scientific, Inc.) following the manufacturer’s instructions. The PCR products were electrophoresed on 2% agarose gels in 0.5 × Tris-borate-EDTA (TBE) (25 mM Tris-borate and 0.5 mM EDTA) followed by staining with ethidium bromide (1 mg/mL), and imaging under UV light.

### DNA-Based O and H Serotyping

DNA-based *E. coli* O and H serotyping (Og- and Hg-typing) were conducted as described previously (Iguchi et al., 2015; Banjo et al., 2018; Iguchi et al., 2020). Briefly, 25 multiplex PCR kits (MP-1 to MP-25) containing 195 primer pairs targeting O-antigen encoding genes and 10 multiplex PCR kits (MP-A to MP-J) containing 52 primer pairs targeting flagellin (H-antigen) encoding genes were used for broad Og- and Hg-typing, respectively. If no PCR product was obtained using any of the multiplex PCR kits, the sample was determined as OgUT (Og untypeable) or HgUT (Hg untypeable).

### Prevalence of Virulence Related Genes

The virulence profiles of *stx1*, *stx2*, and *eae* were screened using PCR. The primers and PCR conditions for *stx1*, *stx2*, and *eae* were previously described by Cebula et al. (1995) and Nguyen et al. (2021), respectively.

### Genome Sequencing and Assembly

DNA for genome sequencing was extracted and purified using the Qiagen Genomic DNA Preparation Kit (Qiagen) or Maxwell^®^ DNA Purification Kit (Promega). Illumina short-read libraries were prepared using 100 ng of extracted DNA using the Nextera DNA Library Prep Kit, and paired-end reads were generated using the MiSeq Reagent Kit (v3-600) on MiSeq (Illumina). MinION long-read libraries were prepared from 300 ng of genomic DNA using the Rapid Barcoding kit (SQK-RBK004) and were sequenced using R9 flow cells (FLO-MIN106) on MinION (Oxford Nanopore Technologies) to produce > 100 Mb of data per strain. The Illumina data were preprocessed using Trimmomatic (v0.36) to remove the adapter and low-quality sequences (Bolger et al., 2014). The quality of the Nanopore sequencing data was assessed using NanoPack (De Coster et al., 2018) a hybrid assembly with the Illumina and Nanopore reads was performed using Unicycler (v0.4.6) with the default parameters (Wick et al., 2017). The sequence data have been deposited in NCBI under the accession number, BioProject PRJDB10561.

### Genome Analysis

Genome sequences were annotated using the online tool DFAST (Tanizawa et al., 2017) and were then visualized using an *in silico* Molecular Cloning software (*in silico* biology, Inc.). Plasmids were identified using PlasmidFinder 2.1 (Carattoli et al., 2014). The family of transposase genes was identified using the online tool ISfinder (Siguier et al., 2006). DNA sequences were aligned using the ClustalW program provided by DNA Data Bank of Japan^1^. A phylogenetic tree was constructed using the neighbor-joining method with 1000 bootstrap replicates in MEGAX (Stecher et al., 2020). Genetic comparison and dot plot analysis of the elements was conducted using GenomeMatcher 3.14 (Ohtsubo et al., 2008).

### Subtyping PCR for the *ter* Operon

The PCR primer sequences used for *ter* operon subtyping are shown in Supplementary Table 4. The thermal conditions for PCR were the same as those of PCR using the universal primers targeting *terA*.

### Multilocus Sequence Typing

Multilocus sequence typing (MLST) was performed using the internal sequences of seven housekeeping genes (*adk, fumC, gyrB*, *icd*, *mdh, purA*, and *recA)* (Wirth et al., 2006). The primers used were described in a previous study (Nguyen et al., 2021). Sequence types (STs) were determined according to the Enterobase MLST website^2^. Concatenated sequences (3,423 bp) of the seven genes were used to construct a phylogenetic tree.

### Minimum Inhibitory Concentration (MIC) of Tellurite

Tellurite resistance was evaluated on Mueller-Hinton agar plates containing twofold serial dilutions of potassium tellurite (K_2_TeO_3_) ranging from 0.125 to 512 μg/mL, according to the general steps of the agar dilution method (Clinical and Laboratory Standards Institutes [CLSI], 2012). Three independent experiments and two replicates were performed for each experiment. For resistant strains, the median log_2_ transformed MIC values of six replicates were used for statistical analysis. The difference in log_2_ transformed MIC was analyzed by Tukey’s *t*-test and statistical significance was set at *p* ≤ 0.05.

## Results

### *terA*-Positive *E. coli* Strains

In total, 106 *terA*-positive *E. coli* strains from humans (26 strains), cattle (34 strains), pigs (6 strains), chickens (8 strains), and deer (32 strains) were collected (see Supplementary Table 3). There was no duplication of the Og:Hg-type in the strains isolated from the same sample. The strains were classified into 78 Og:Hg types, including an OgUT:Hg2 strain. *terA*-positive *E. coli* strains contained *eae*-positive STEC (positive for either or both *stx1* and *stx2*) (42 strains), *eae*-negative STEC (28 strains), enteropathogenic *E. coli* (EPEC) (negative for both *stx1* and *stx2* and positive for *eae*) (4 strains), and others (all negative for *stx1*, *stx2*, and *eae*) (32 strains).

### Subtypes of *ter* Operon

**FIGURE 1:**
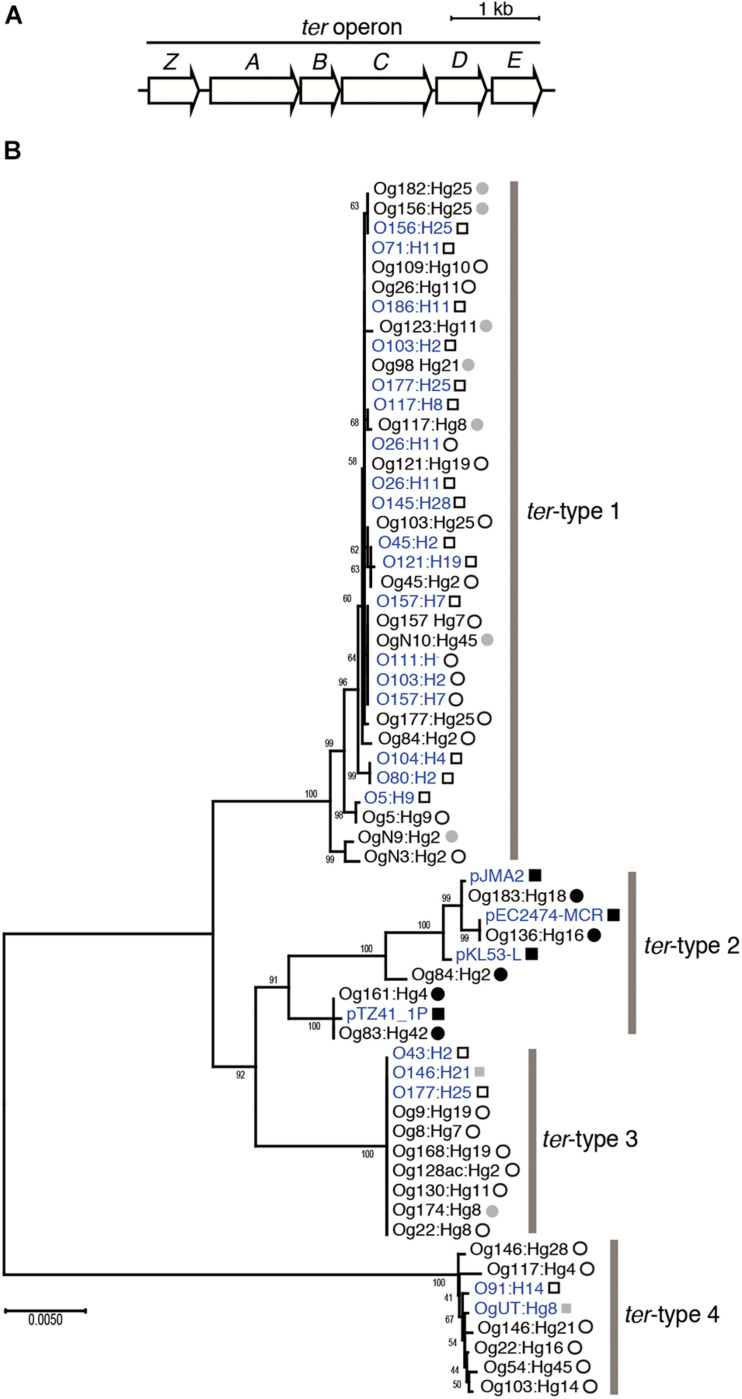
Structure of the *ter* operon **(A)**. Phylogenetic tree of the *ter* operon **(B)**. The tree was created based on the *ter* operon sequences (4,546–4,551 bp) from 34 *Escherichia coli* strains used in this study, and 27 *E. coli* genomes registered in DNA database. Four of them were isolated in Japan and the remaining 23 were isolated outside Japan. Strains isolated in Japan and outside Japan are indicated by circles and squares, respectively. Open and fulfilled circles or squares indicate that the *ter* operon is located on the chromosome and on the plasmid, respectively. Those whose location is unknown are shown in gray. DNA sequences obtained from the DNA database (GenBank/ENA/DDBJ) are shown in blue color. Bootstrap values are displayed at the branching points.

First, we determined the draft genomes of 34 representative strains using the MiSeq system, which were randomly selected after sorting out so that the Og:Hg types did not overlap (Supplementary Table 3) and extracted all *ter* operons (4,546 − 4,551 bp) containing six genes *terZ*, *terA*, *terB*, *terC*, *terD*, and *terE* (Figure 1A). When a phylogenetic tree was created using the *ter* operon sequences, it became clear that four groups were formed, named *ter*-type 1–4 (Figure 1B). In particular, the nucleotide sequences of types 1, 3, and 4 were highly conserved within their respective groups. The phylogenetic trees using the sequences of each gene of 34 strains, *terZ* (582 bp), *terA* (1,158 bp), *terB* (456 bp), *terC* (1,041 bp), *terD* (579 bp), and *terE* (576 bp) showed that type-3 and type-4 formed independent groups for all genes, whereas type-1 and type-2 roughly formed their respective groups for *terZ*, *terA*, *terD*, and *terE* (Supplementary Figure 1). When a phylogenetic tree was created by adding 13 types of *ter* operons possessed by bacteria other than *E. coli* (Supplementary Table 2), three operons from *Yersinia pseudotuberculosis*, *Salmonella enterica*, and *Klebsiella pneumoniae* belonged to type-2, operons from seven *Enterobacteriaceae* genera formed an additional clade inside the group of *E. coli*-derived operons but no *E. coli* strain belonged to this clade, and the remaining three from *Proteus mirabilis*, *Yersinia pestis*, and *Pseudomonas aeruginosa* were distant from the *E. coli*-derived group (Supplementary Figure 2).

### Comparison of Elements Carrying *ter* Operons

To analyze the structure of the *ter* operon as well as the surrounding region in detail, complete or almost complete genome sequences were obtained from the short- and long-read sequences of the 26 strains (Supplementary Table 3). When the attributes of *ter* operons were confirmed from the surrounding sequence information, the operons classified as *ter*-type 1, 3, and 4 were located on the chromosomes, whereas all operons classified as *ter*-type 2 were located on plasmids (Figure 1 and Supplementary Figure 2).

**FIGURE 2:**
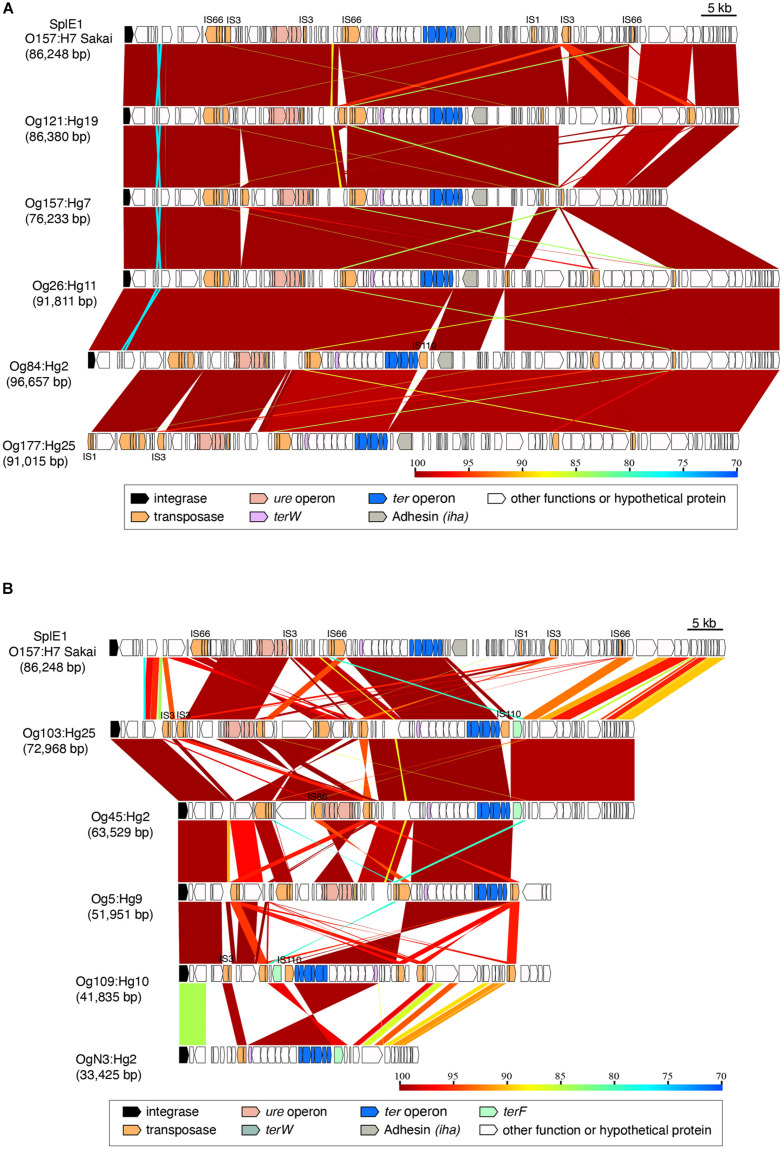
Genomic comparison of the integrating elements carrying *ter*-type 1. **(A)** Comparison between SplE1 and SplE1-like elements; **(B)** comparison among SplE1 with SplE1-like elements and unknown elements. The approximate sizes of the elements are indicated in parentheses. Genes are colored based on their functional characteristics as described in the legend. The nucleotide sequence identities between the elements (cutoff ≥ 70% identity) are indicated by color shading according to the scale shown at the bottom of the figure.

The elements carrying *ter*-type 1 from Og157:Hg7, Og121:Hg19, Og26:Hg11, Og84:Hg2, and Og177:Hg25 (76,233–96,657 bp) were highly conserved with SpLE1 in the O157:H7 Sakai strain (Figure 2A and Supplementary Figure 3). In addition to the *ter* operon, these elements also carried the conserved integrase gene, the *ure* operon, *iha*, and some transposase genes including the IS66, IS3, and IS110 families; however, the integrase gene was not found in Og177:Hg25. The elements carrying *ter*-type 1 in Og103:Hg25, Og45:Hg2, Og5:Hg9, Og109:Hg10, and OgN3:Hg2 were different from those in the SpLE1 group. Although these elements were conserved in the *ter* operon and in some regions, there were many indels, inversions, and recombination (Figure 2B and Supplementary Figure 3).

**FIGURE 3:**
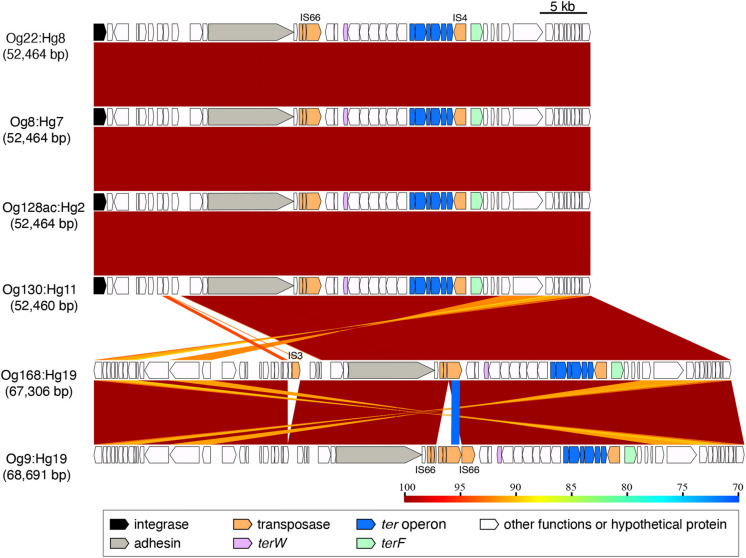
Genomic comparison of the integrating elements carrying *ter*-type 3. The approximate sizes of the elements are indicated in parentheses. The genes are colored based on the functional characteristics as described in the figure legend. The nucleotide sequence identities between the elements (cutoff ≥ 70% identity) are indicated by color shading according to the scale shown at the bottom of the figure.

The elements carrying *ter*-type 3 from Og22:Hg8, Og8:Hg7, Og128ac:Hg2, Og130:Hg11, Og168:Hg19, and Og9:Hg19 (52,460–68,691 bp) were almost identical, except for the upstream region in Og168:Hg19 and Og9:Hg19 (Figure 3 and Supplementary Figure 3). It included an adhesin gene and some transposase genes, including the IS66 and IS4 families.

**FIGURE 4:**
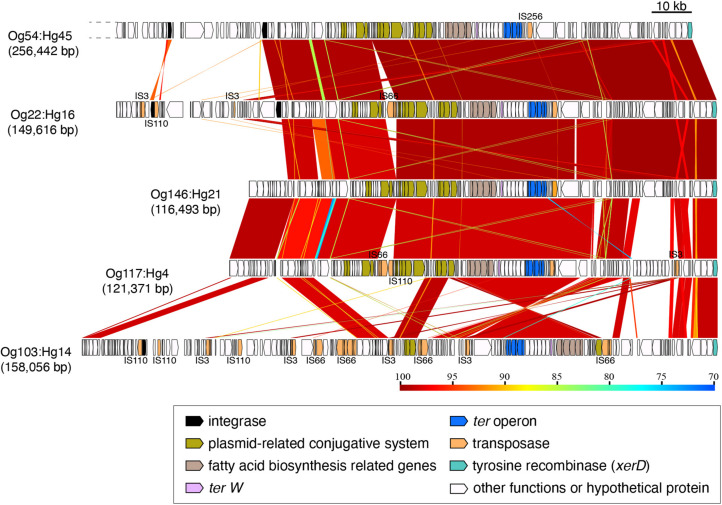
Genomic comparison of the integrating elements carrying *ter*-type 4. Dashed lines indicate cutoff sequences. The approximate sizes of the elements are indicated in parentheses. Genes are colored based on their functional characteristics as described in the figure legend. The nucleotide sequence identities between the elements (cutoff ≥ 70% identity) are indicated by color shading according to the scale shown at the bottom of the figure.

The *ter*-type 4 operons from Og54:Hg45, Og22:Hg16, Og146:Hg21, Og117:Hg4, and Og103:Hg14 were located on elements of different sizes ranging from 116,493 to 256,442 bp (Figure 4). However, the downstream regions of about 100 kb including the *ter* operon, were conserved, except for that of Og103:Hg14 (Figure 4 and Supplementary Figure 3). Conserved regions contained a plasmid-conjugative system (*tra* genes), a fatty acid biosynthesis gene cluster, site-specific tyrosine recombinase genes, and some transposase genes, including the IS256 family.

**FIGURE 5:**
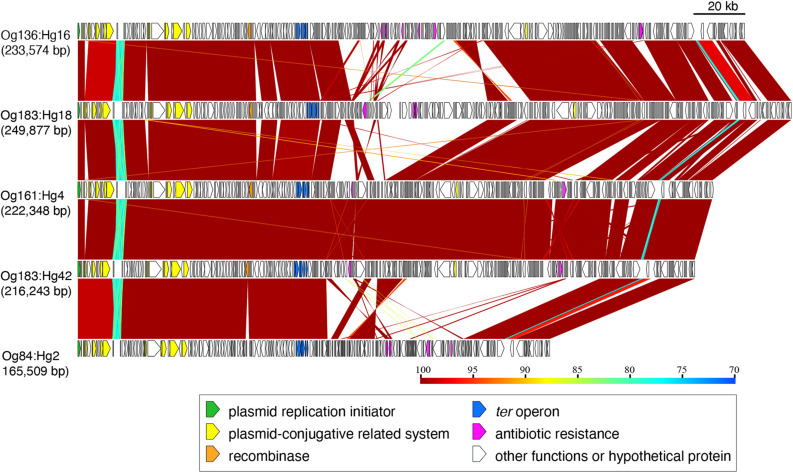
Genomic comparison of the plasmids carrying *ter*-type 2. The plasmid sizes are indicated in parentheses. Genes are colored based on their functional characteristics as described in the figure legend. The nucleotide sequence identities between the elements (cutoff ≥ 70% identity) are indicated by color shading according to the scale shown at the bottom of the figure.

The operon of *ter*-type 2 from Og136:Hg16, Og183:Hg18, Og161:Hg4, Og183:Hg42, and Og84:Hg2 was located on IncHI2 plasmids, including the conjugative system (*tra* genes) and some antibiotic resistant genes [*aadA*, *aph* (3’), *bla*, *ble*, *cml*, *eptA*, and *tetA*], with sizes ranging from 165,509 to 249,877 bp (Figure 5 and Supplementary Figure 3). Except for the central regions downstream of the *ter* operons, where multiple recombination and inversions were confirmed, these plasmids roughly retained homologous gene structures. Based on the coverage of genomic sequences, the copy number of plasmids carrying the *ter*-operons was approximately the same as that of the chromosomes (1 vs. 0.85–1.74).

All *ter* operons used in this study, which were extracted from the genomes of strains other than *E. coli*, were located on plasmids, except for a *Salmonella*-derived *ter*-type 2 operon, and *Proteus*- and *Yersinia*-derived operons, which are on the chromosome (Supplementary Figure 2).

### The Insertion Sites of Integrating Elements Carrying the *ter* Operon on the Chromosome

**FIGURE 6:**
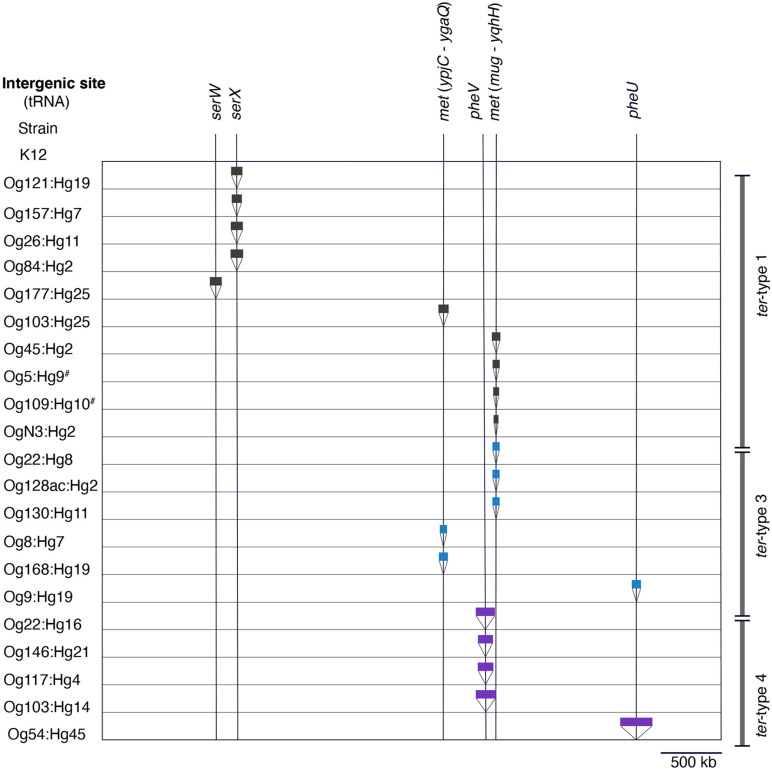
Insertion sites of elements carrying the *ter* operon in the chromosome. The sites are based on the positions of tRNA in the genome of the K-12 MG1655 strain. Black, blue, and purple bars indicate elements carrying *ter*-type 1, 3, and 4, respectively. The width of the bars is proportionate with the element size. Hash key indicates the absence of tRNA at the site.

The insertion sites of elements carrying the *ter* operon are summarized in Figure 6. tRNA^SerX^ and tRNA^PheV^ were the only insertion sites of elements carrying *ter*-type 1 and 4 operons, respectively. Two positions of tRNA^Met^ (*ypjC*–*ygaQ* and *mug*–*yqhH*) were the insertion sites for the elements of the *ter*-type 1 and 3 operons. Although the types of elements carrying the *ter*-type 1 and 3 operons were different, a homology (98% amino acid sequence) was confirmed in the integrase genes located on the elements inserted in tRNA^Met^ (Supplementary Figure 4). Similarly, the nucleotide sequences of the integrase in the element carrying *ter*-type 4 (Og54:Hg45 and Og22:Hg16 strain) were highly homologous (upper 97% amino acid sequence) with the integrases in the other integrating elements or the locus of enterocyte effacement that was inserted in the tRNA^Phe^ positions in seven major STEC genomes (Supplementary Table 1) (data not presented). In addition, the element carrying the *ter*-type 1 operon of Og177:Hg25 was inserted into tRNA^SerW^, the element carrying *the ter*-type 3 operon of Og9:Hg19 was inserted into tRNA^PheU^, and the element carrying the *ter*-type 4 operon of Og54:Hg45 was in tRNA^PheU^. Although tRNA was absent in the insertion site of the *ter* operon-carrying elements in the Og5:Hg9 and Og109:Hg10 strains, a corresponding region surrounding the indicated tRNA was present in each strain (Supplementary Figure 5). There was an element other than the *ter* operon carrier inserted in the tRNA^PheU^ position of the Og9:Hg19 strain (Supplementary Figure 5).

### PCR for *ter* Operon Subtyping

Based on the sequences of the *ter* operons belonging to four subtypes, we designed type-specific PCR primers, and confirmed that these primers could specifically determine each type (Supplementary Table 4 and Supplementary Figure 6). Using PCR, all 106 *ter*-positive strains tested were successfully classified into 4 *ter*-types: type 1 (*n* = 66), type 2 (*n* = 13), type 3 (*n* = 8), and type 4 (*n* = 17), except for two strains belonging to Og84:Hg2, which were positive for both *ter*-type 1 and 2 (Supplementary Table 3). All *eae*-positive strains, including seven major STEC serotype strains, were positive for *ter*-type 1 (*n* = 46), whereas *eae*-negative *E. coli* strains, including STEC and non-STEC, were positive for *ter*-type 2 (*n* = 13), type 3 (*n* = 8), and type 4 (*n* = 17), in addition to *ter*-type 1 (*n* = 21) (Supplementary Table 3).

### Distribution of *ter*-Types in *Escherichia coli* Linages

**FIGURE 7:**
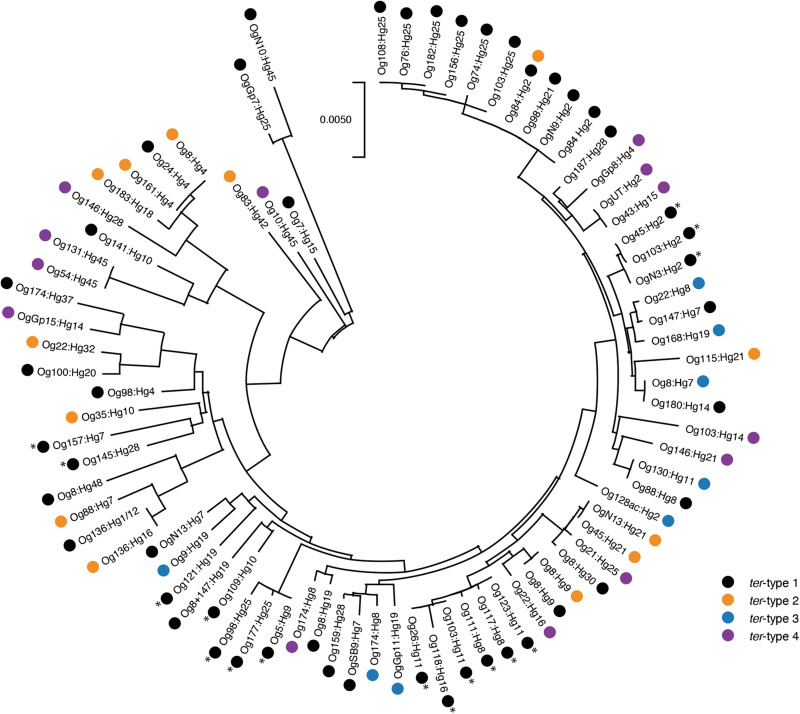
Phylogenetic tree of 80 *terA*-positive *Escherichia coli* strains. The tree was built based on the internal concatenated sequences of seven housekeeping genes (3,423 bp). The asterisk indicates *eae*-positive strains.

The phylogenetic tree of 80 *terA*-positive *E. coli* strains (one representative strain of the same O:H genotype and the same *ter*-type was selected from 106 strains) is shown in Figure 7. Overall, four *ter*-types were distributed in various lineages of *E. coli* including A, B1, B2, E, D, A × B1, and ABD (Supplementary Table 3), although some strains with the same *ter*-type formed several small clades.

### Tellurite Resistance

**FIGURE 8:**
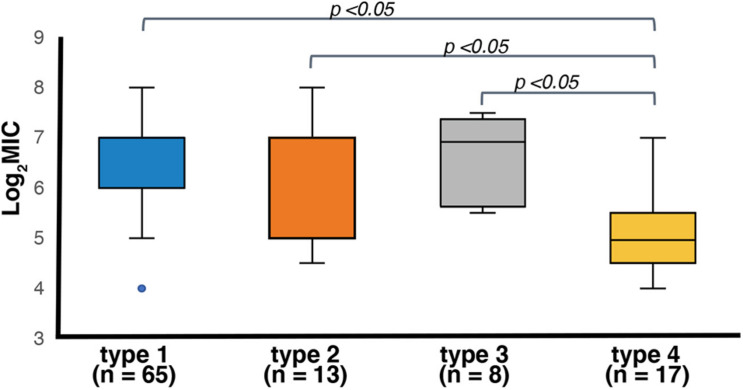
Box plots of log_2_ transformed MIC distributions of potassium tellurite (K_2_TeO_3_) based on the *ter* operon type. Boxes indicate the 25th, 50th, and 75th percentiles. Whiskers indicate the lowest and highest data point. Outliers are shown as blue circles. *P*-values of statistically significant differences are shown (*p* < 0.05).

The 106 *ter*-positive *E. coli* strains showed MICs ranging from 16 to 256 μg/mL of potassium tellurite (Supplementary Table 5), except for a *ter*-type 1 strain (KAP39, Og98:Hg4), which was tellurite-sensitive. The genome sequence of this strain had an IS5 inserted on the *terZ* due to which the gene was disrupted (Supplementary Figure 7). Based on the *ter* subtype, seventy-five percent of the strains were classified as *ter*-type 1, 2, and 3 were resistant to potassium tellurite concentrations of 64–128 μg/mL or higher, whereas only one strain (6%) in *ter*-type 4 was resistant to 64–128 μg/mL (Supplementary Table 5). There was no significant difference in the resistance among *ter*-type 1, 2, and 3 groups, whereas the resistance activity of *the ter*-type 4 group was significantly lower than that of the other three types (*p* < 0.05) (Figure 8).

## Discussion

The gene composition and sequence homology of the *ter* operon carrier elements on the chromosomes were roughly conserved for each of the *ter*-types, 1, 3, and 4 (Supplementary Figure 3), and strains belonging to different phylogenetic lineages carried the same types of elements (Figure 7), suggesting that at least three different types of chromosomal elements carrying the *ter* operons have spread horizontally within *E. coli* species. Six tRNA genes on the chromosome were confirmed as insertion sites for these elements, and were classified into three tRNA types: tRNA^Ser^, tRNA^Met^, and tRNA^Phe^. The integrase genes on the elements carrying the *ter*-type 1 operon were divided into two types according to the sequence homology: elements with one type of integrase gene were inserted into tRNA^Ser^ (as seen in strains belonging to O157:H7, and so on) and elements with the other type of integrase gene were inserted into tRNA^Met^ (as seen in strains belonging to O103:H25, and so on) (Figures 2, 6). The latter integrase gene showed high homology with that of *ter*-type 3 operon-carrying elements (Supplementary Figure 4), and all elements with the *ter*-type 3 operon were also inserted into tRNA^Met^, except for one strain (A150083 of Og9:Hg19) (Figure 6). Although the integrase gene was found only in two of the five *ter*-type 4 operon carrier elements used in this study, these two were conserved and highly homologous with the integrase in other elements of other strains that were all inserted into tRNA^Phe^. These results suggest that the insertion site of these elements on the chromosome depends strongly on the integrase genes and not on the basic structure of the elements. Transposases such as IS66 and IS256 were located upstream and/or downstream of the *ter* operons, and especially in some *ter*-type 1 operons, they coincided with the recombination and inversion junction points, suggesting that IS transposase was involved in the transfer of small fragments, including *ter* operons, to other elements.

The *ter*-type 2 operons were located on the IncHI2 plasmids of *E. coli* strains, and were also found on the IncHI2 plasmids carried by *Y. pseudotuberculosis* and *K. pneumoniae* (Supplementary Figure 2), suggesting that this type of operon was horizontally transferred in *E. coli* as well as within the *Enterobacteriaceae* through plasmids. The conjugative IncHI2 plasmid is widely found in *Enterobacteriaceae* and is known to mediate various drug resistance genes (Herrera-León et al., 2011; Roberts et al., 2020). In fact, the plasmids found to harbor the *ter*-type 2 operons in this study carried three or more drug resistance genes (Figure 5). In this study, most of the *ter*-type 2 operons were found in isolates from chickens (47%, 7/15). In recent years, large amounts of antibacterial drugs have been used in broiler production, and the resulting emergence and spread of drug-resistant bacteria has become a global public health concern (Nhung et al., 2017; Mehdi et al., 2018; Yang et al., 2019). The IncHI2 plasmid carrying drug resistance genes may have thus been selected in the gastrointestinal tract of chickens after many years of antibacterial drug use, and the *ter* operon coexisting on the plasmid was co-selected simultaneously.

All *E. coli* strains used in this study were isolated in Japan. However, the sequences of the *ter* operons obtained from the DNA database used in Figure 1 originated from *E. coli* isolated outside Japan (14 strains belonging to type 1, 4 strains to type 2, 3 strains to type 3, and 2 strains to type 4), except for 4 strains belonging to type 1 (O157:H7, O103:H2, 0111: H-, and O26:H11) (see Supplementary Table 1). These results suggested that the characteristics of the *ter* operon revealed in this study were not only those found in *E. coli* isolated in Japan, but also common to *E. coli* isolated outside Japan.

Strains carrying the *ter*-type 4 operon showed significantly lower MICs of potassium tellurite compared to the other three types. The function of Ter proteins has been suggested to involve their mutual association, as they interact at the interface of the inner plasma membrane and the cytosol (Valkovicova et al., 2013; Turkovicova et al., 2016). The nucleotide and amino acid sequences of genes on the *ter*-type 4 operon were significantly different from those of the other three types (Supplementary Figure 1), suggesting that these differences directly affected the functionality of Ter proteins. Most of the *ter*-type 4 operons were found in isolates from wild deer (94%, 16/17). Of the isolation sources used in this study, deer were the only animals from the wild environment, and their feces were collected from a wide area ranging from Hokkaido in the north, to Kyushu in the south of Japan. In addition to tellurite resistance, the *ter* operon is also involved in resistance to infection by various bacteriophages and to membrane-pore forming colicins (Whelan et al., 1995, 1997; Alonso et al., 2000), tolerance to oxidative stresses (Valková et al., 2007), resistance to phagocytosis by macrophages (Ponnusamy and Clinkenbeard, 2015), the ability of cells to adhere to epithelial cells *in vitro* (Yin et al., 2009), and filamentous bacterial cellular morphology (Whelan et al., 1997). In addition, a *ter* homolog from *Clostridium acetobutylicum* showed resistance to methyl methane sulfonate and mitomycin C when expressed in an *E. coli recA* mutant (Azeddoug and Reysset, 1994). Bacterial acquisition of the *ter* operon may thus be beneficial for colonization of the host intestinal tract in the presence of various stresses and attacks. Although the reason for the strong relationship between the *ter* type-4 operon and deer is currently unknown, food and water in the wild environment, and the wild deer gut microbiome formed by their effects, may select strains carrying this type of *ter*-operon.

The tellurite-resistant phenotypes were variable within each type. For example, five strains of Og177: Hg25 with the *ter*-type 1 operon showed MICs of potassium tellurite ranging from 16 to 128 μg/mL. Furthermore, the amino acid sequences of the *ter*-type 1 operon genes of major STEC O26:H11 (11,368 strain) was identical to those of Og121:Hg19 (TS23_1 strain) (data not show). However, the resistance level of the latter strain (16–32 μg/mL of potassium tellurite) was roughly lower than that of the former (64–128 μg/mL). A previous study has also shown that even O157:H7 serotype strains differ in their MICs of potassium tellurite (64–1,024 μg/mL) (Taylor et al., 2002). The above results indicate that the *ter* operon as well as other genetic factors influence tellurite resistance.

## Conclusion

This study compared the details of *ter* operons responsible for tellurite resistance among various serotypes and sources of *E. coli*. Comparative analysis clearly distinguished the *ter* operon sequences into four subtypes, namely, *ter*-type 1–4 operons. The amino acid sequences of the *ter*-type 4 operon genes were distant from those of the other types, further, strains with the *ter*-type 4 operons showed significantly lower MICs compared to the other types. Furthermore, genomic analysis using long reads indicated the structural details of the integrating elements carrying the *ter* operons and insertion sites on the chromosome. Genomic comparison and phylogenetic analysis showed that at least three highly conserved elements were spread horizontally within *E. coli* species. Type 2 was found to be located on plasmids distributed in *E. coli* as well as in some *Enterobacteriaceae* species. The *ter*-type was also closely related to the source of isolation, the cause of which was unknown; however, types 2 and 4 were associated with chickens and deer, respectively. This study provided new insights related not only to genetic characteristics of the *ter* operons in *E. coli*, but also to phenotypic and ecological differences that may be related to them. In the future, by clarifying the unknown function of the Ter proteins, it is expected that the relationship between the diversity of the *ter* operon and the phenotypic and ecological characteristics revealed in this study will be clarified.

## Data Availability Statement

The datasets presented in this study can be found in online repositories. The names of the repository/repositories and accession number(s) can be found in the article/Supplementary Material.

## Author Contributions

TN, AI, and SI conceived and designed the experiments. TN performed the experiments. TN, TK, and TT analyzed the data. TN and AI contributed to reagents, materials, analysis tools, critical revision of the manuscript for important intellectual content, and wrote the manuscript. All authors contributed to the article and approved the submitted version.

## Conflict of Interest

The authors declare that the research was conducted in the absence of any commercial or financial relationships that could be construed as a potential conflict of interest.

## Footnotes

1 https://clustalw.ddbj.nig.ac.jp

2 http://enterobase.warwick.ac.uk/species/ecoli/allele_st_search
