# Supplementary material for: Diversity of the Tellurite Resistance Gene Operon in Escherichia coli
Source: Front Microbiol. 2021 May 28;12:681175. doi: 10.3389/fmicb.2021.681175 (PMC8193136; doi:10.3389/fmicb.2021.681175)
Supplement: Supplementary file 1 [file Data_Sheet_1.pdf]

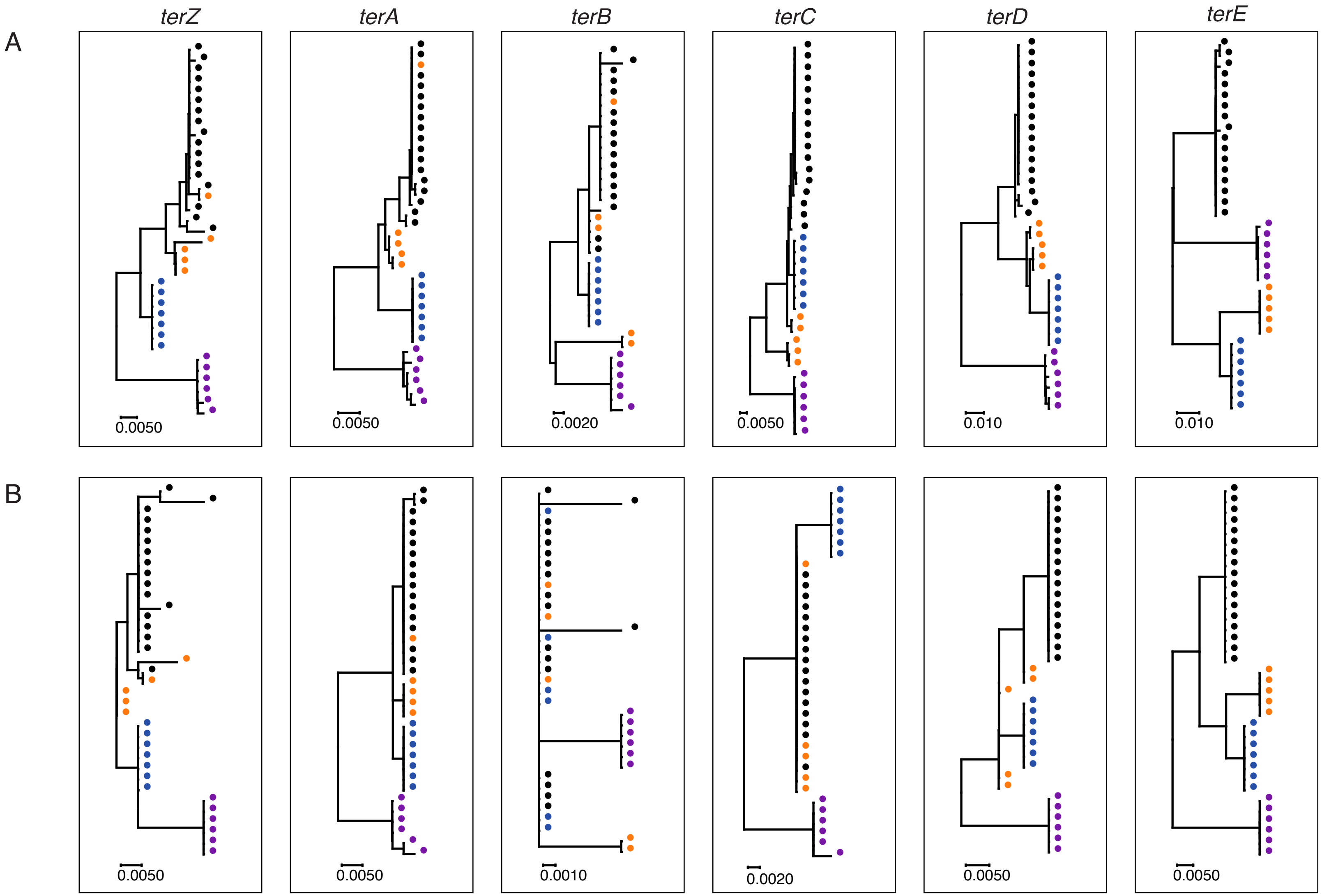

Supplementary Figure 1. Phylogenetic trees of nucleotide (A) and amino acid sequences (B) of each gene of the *ter* operons from 34 *E. coli* strains used in this study (Table S3). Black, orange, blue, and purple circular dots are represented for strains carrying *ter*-type 1, 2, 3, and 4, respectively. The scale bars indicate number of nucleotides (A) or amino acids (B) substitution per site.

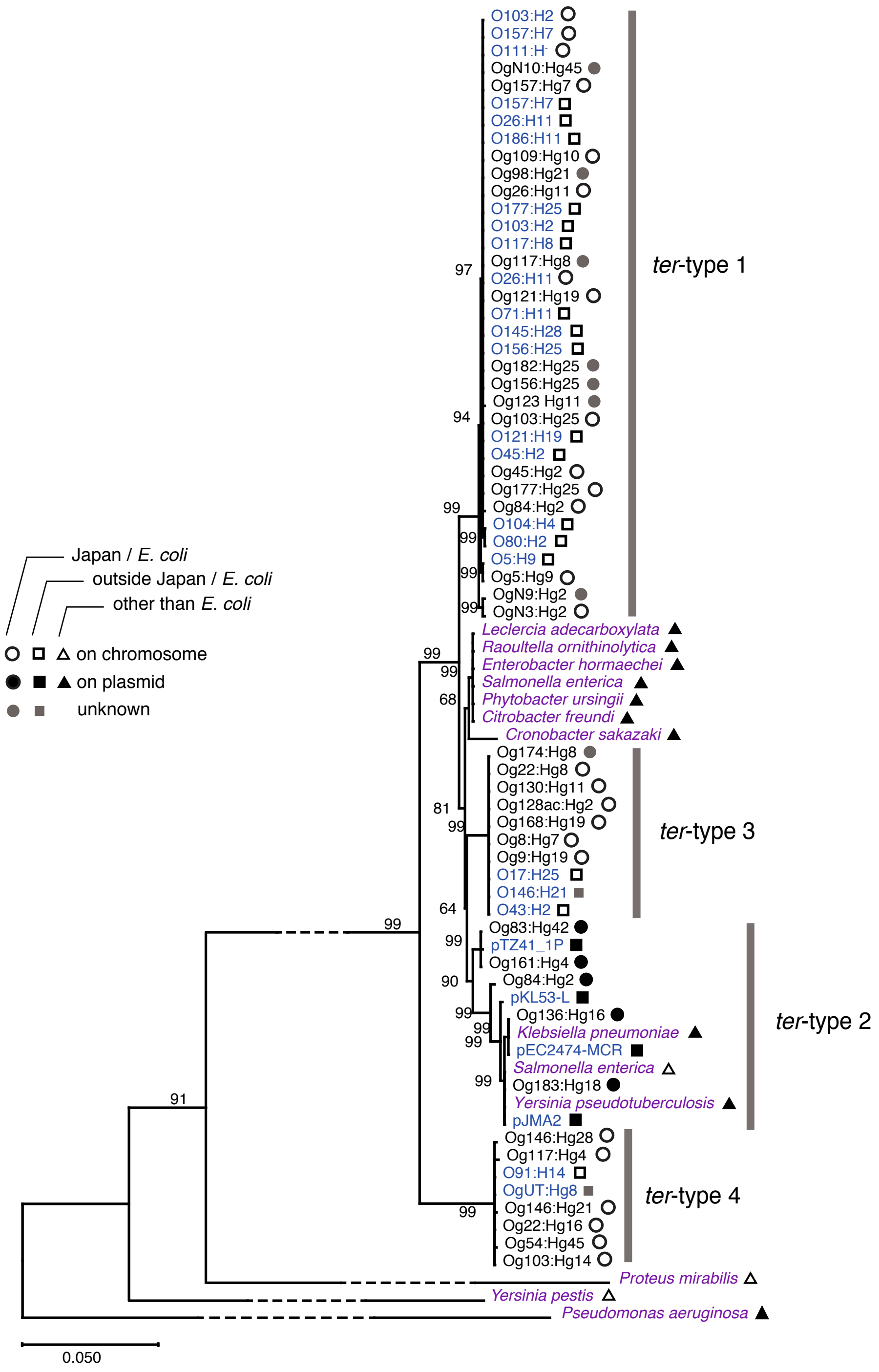

Supplementary Figure 2. Phylogenetic tree of the *ter* operon from *E. coli* (61 strains) and other species (13 strains). Strain sequences obtained from DNA database (GenBank/ENA/DDBJ) of *E. coli* and other species are shown in the blue and purple colors, respectively. The dash lines indicate a shortened distance from the original tree. Bootstrap values are displayed at the branching points.

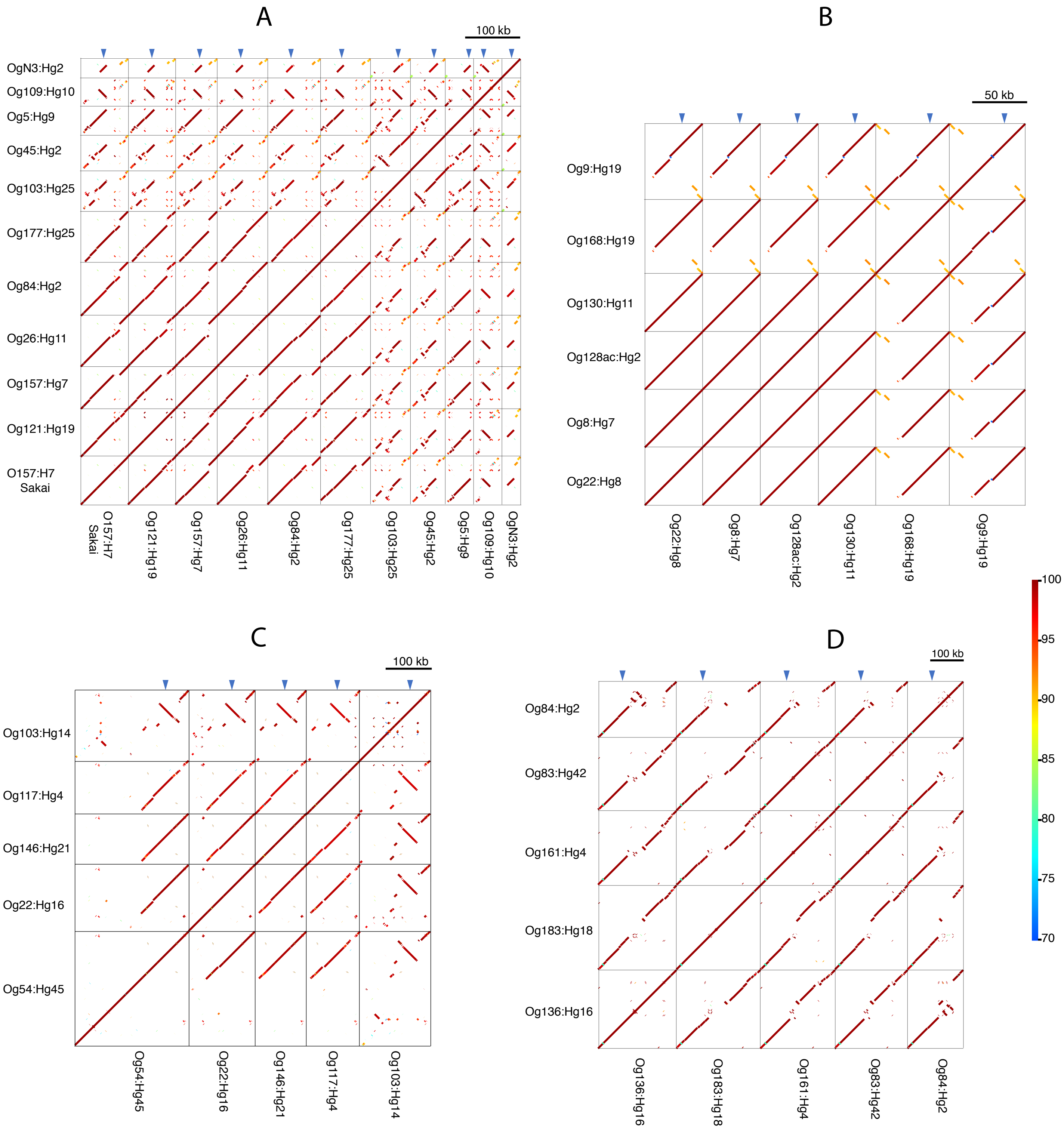

Supplementary Figure 3. Dot-plot matrices of integrating elements carrying *ter*-type 1 (A), *ter*-type 3 (B), *ter*-type 4 (C), and plasmid carrying *ter*-type 2 (D). The position of the *ter* operon is pointed by a blue triangle on the horizontal line for each strain. The nucleotide identities between elements (cutoff ≥ 70% identity) are indicating by color shading according to the scale shown at the right site of the figure.

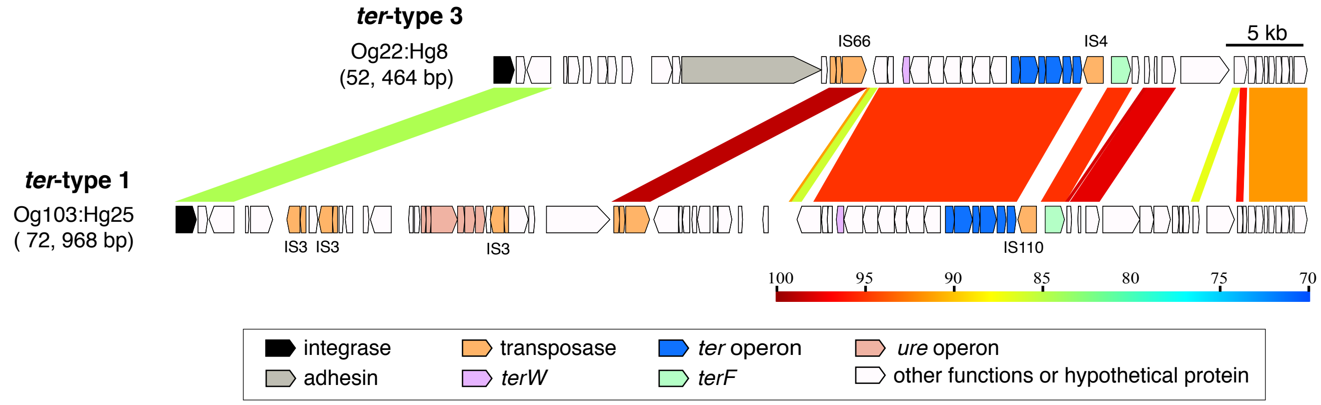

Supplementary Figure 4. Genomic comparison between element carrying *ter*-type 3 (Og22:Hg8 strain) and *ter*-type 1 (Og103:Hg25 strain). The approximate sizes of the elements are indicated in parentheses. Gene are colored based on functional characteristics as described in the figure legend. The nucleotide sequence identities between elements (cutoff 70% identity) are indicating by color shading according to the scale shown at the bottom of the figure.

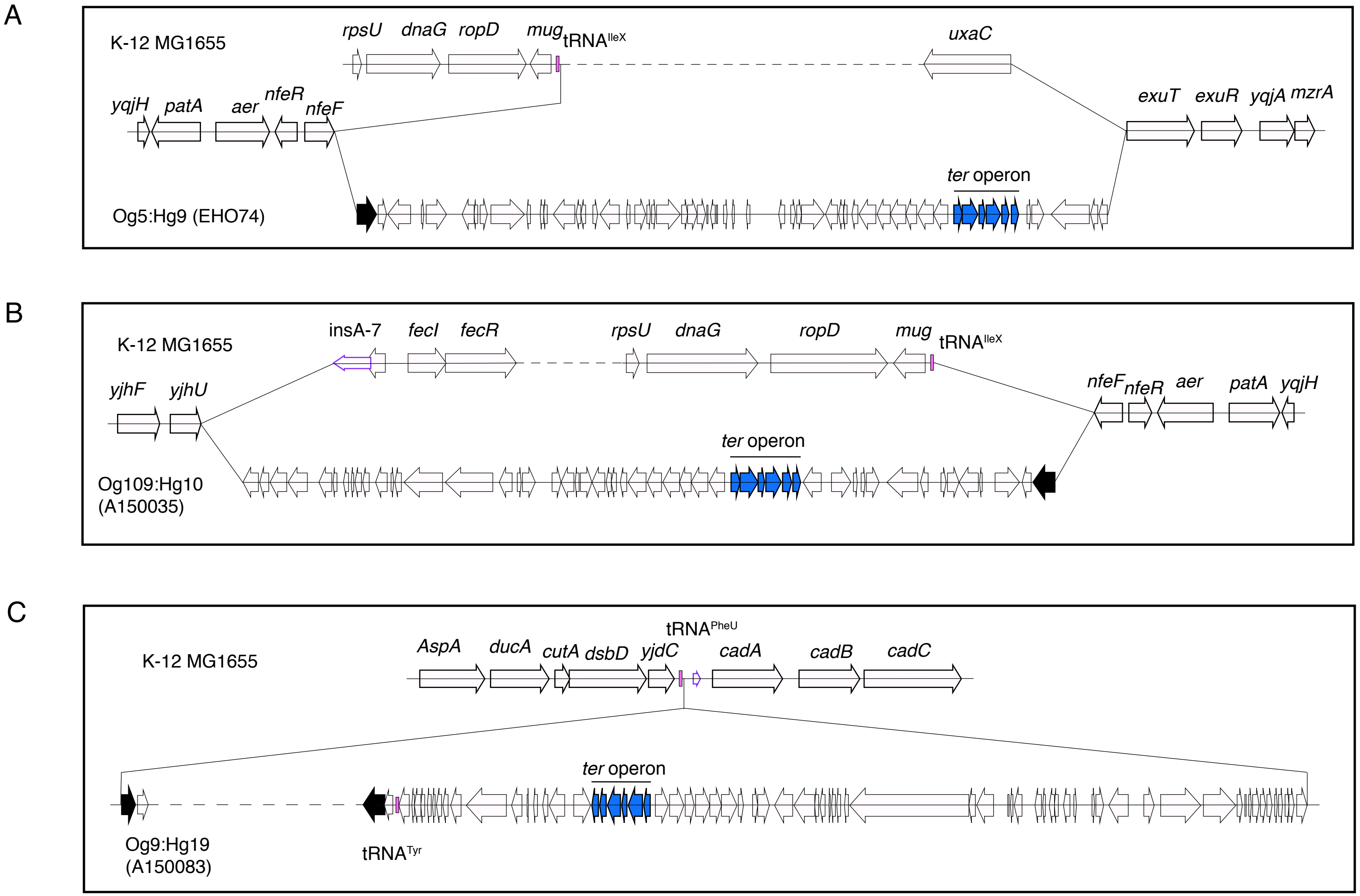

Supplementary Figure 5. The insertion sites of elements carrying *ter*-operon of Og5:Hg9 (A), Og109:Hg10 (B), and Og9:Hg19 (C) strains in comparison with K12-MG1655 genome. Black, blue, purple, and white arrows are represented for integrase-encoding gene, *ter* operon genes, pseudo genes, and other genes or hypothetical coding sequences, respectively. Dash lines indicate coding sequence are not shown.

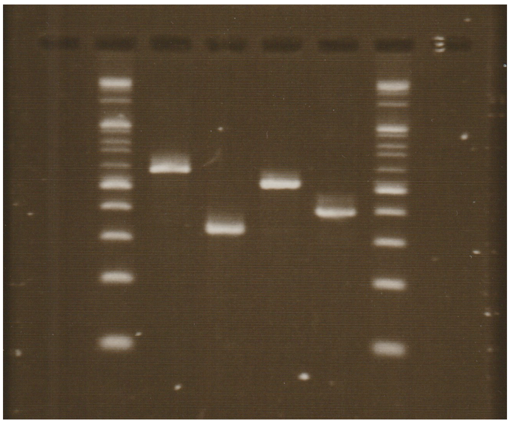

Supplementary Figure 6. Gel image of the PCR products of *ter*-type 1, 2, 3, and 4. Lanes at the both ends are sizes markers (100-bp DNA ladder; Shinkoseiki.co.ltd.).

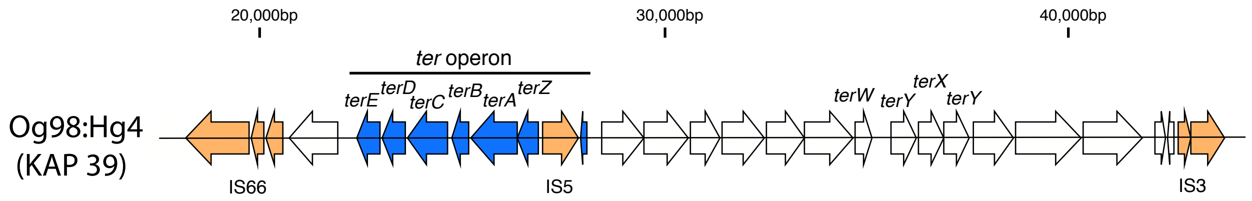

Supplementary Figure 7. The structure of the *ter* operon and surrounding regions of Og98:Hg4 (KAP39) strain. White arrows indicate other genes or hypothetical proteins.
